# Improved detection of microbiological pathogens: role of partner and non-governmental organizations

**DOI:** 10.1186/s12879-021-05999-8

**Published:** 2021-03-25

**Authors:** Michael Owusu, Bernard Nkrumah, Godfred Acheampong, Ebenezer Kofi Mensah, Abass Abdul-Karim Komei, Festus Kofi Sroda, Sambian David, Shannon Emery, Lucy Maryogo Robinson, Kwame Asante, David Opare

**Affiliations:** 1 Centre for Health Systems Strengthening, Kumasi, Ghana; 2 Department of Medical Diagnostics, Kwame Nkrumah University of Science and Technology, Kumasi, Ghana; 3 African Field Epidemiology Network, Accra, Ghana; 4 Sekondi Public Health Laboratory, Ghana Health Service, Sekondi, Ghana; 5 Tamale Public Health Laboratory, Ghana Health Service, Tamale, Ghana; 6 Kumasi Public Health Laboratory, Ghana Health Service, Kumasi, Ghana; 7 Association of Public Health Laboratories, Silver Springs, MD USA; 8 National Public Health and Reference Laboratory, Accra, Ghana

**Keywords:** Pathogens, Public health laboratories, Detection, Non-governmental organization

## Abstract

**Background:**

Proper detection of disease-causing organisms is very critical in controlling the course of outbreaks and avoiding large-scale epidemics. Nonetheless, availability of resources to address these gaps have been difficult due to limited funding. This report sought to highlight the importance of in-country partners and non-governmental organizations in improving detection of microbiological organisms in Ghanaian Public Health Laboratories (PHLs).

**Methods/context:**

This study was conducted between June, 2018 to August, 2019. U. S CDC engaged the Centre for Health Systems Strengthening (CfHSS) through the Association of Public Health Laboratories to design and implement strategies for strengthening three PHLs in Ghana. An assessment of the three PHLs was done using the WHO/CDS/CSR/ISR/2001.2 assessment tool. Based on findings from the assessments, partner organizations (CfHSS/APHL/CDC) serviced and procured microbiological equipment, laboratory reagents and logistics. CfHSS provided in-house mentoring and consultants to assist with capacity building in detection of epidemic-prone infectious pathogens by performing microbiological cultures and antimicrobial susceptibility tests.

**Results:**

A total of 3902 samples were tested: blood (1107), urine (1742), stool (249) and cerebrospinal fluid (CSF) (804). All-inclusive, 593 pathogenic bacteria were isolated from blood cultures (70; 11.8%); urine cultures (356; 60%); stool cultures (19; 3.2%) and from CSF samples (148; 25%). The most predominant pathogens isolated from blood, urine and stool were *Staphylococcus aureus* (22/70; 31%), *Escherichia coli* (153/356; 43%) and *Vibrio parahaemolyticus* (5/19; 26.3%), respectively. In CSF samples, *Streptococcus pneumoniae* was the most frequent pathogen detected (80/148; 54.1%). New bacterial species such as *Pastuerella pneumotropica, Klebsiella oxytoca, Vibrio parahaemolyticus,* and *Halfnia alvei* were also identified with the aid of Analytical Profile Index (API) kits that were introduced as part of this implementation. *Streptococcus pneumoniae* and *Neisseria meningitidis* detections in CSF were highest during the hot dry season. Antimicrobial susceptibility test revealed high rate of *S. aureus, K. pneumoniae* and *E. coli* resistance to gentamicin (35–55%). In urine, *E. coli* was highly resistant to ciprofloxacin (39.2%) and ampicillin (34%).

**Conclusion:**

Detection of epidemic-prone pathogens can be greatly improved if laboratory capacity is strengthened. In-country partner organizations are encouraged to support this move to ensure accurate diagnosis of diseases and correct antimicrobial testing.

## Background

Globally, the fight against infectious diseases is still a great force to reckon with. The ability of disease-causing organisms to spread beyond national and international borders means an infectious disease threat anywhere is a threat everywhere [1]. Thus, every country has a role to play in making the world safer from epidemics by strengthening its capacity to prevent, detect in timely manner and respond effectively to current and emerging health threats. Key health threats that could likely pose danger to human lives include increasing trend of antimicrobial resistance, zoonotic diseases, biosafety and biosecurity, weak laboratory and surveillance systems and poor work force development.

Addressing the threats of zoonosis, antimicrobial resistance, biosafety and biosecurity begins with detection of aetiological agents involved in disease outbreaks and infections. Timely and accurate detection and reporting of infectious disease outbreaks and events are critical to controlling the course of outbreaks and avoiding large-scale epidemics. Detection of microbial pathogens also enables the performance, reporting and surveillance of antimicrobial resistant microbial organisms.

Antimicrobial resistance is one of the biggest threats to global health [2]. According to WHO, there are 12 families of resistant bacteria which pose the greatest threat to human health, and these are termed priority pathogens [3]. These bacteria are further categorized into Priorities 1 (critical), 2 (high) and 3 (medium). Examples of priority 1 pathogens include Carbapenem-resistant *Acinetobacter baumannii*, Carbapenem-resistant *Pseudomonas aeruginosa* and Carbapenem-resistant, ESBL-producing Enterobacteriaceae. Priority 2 pathogens include Vancomycin-resistant *Enterococcus faecium*, Methicillin and Vancomycin-resistant *Staphylococcus aureus*, Clarithromycin-resistant *Helicobacter pylori*, Fluoroquinolone-resistant *Salmonella* spp., Fluoroquinolone-resistant *Campylobacter* spp., and Cephalosporin-resistant, fluoroquinolone-resistant *Neisseria gonorrhoeae*. Other bacteria such as Penincillin-non-susceptible *Streptococcus pneumoniae*, Ampicillin-resistant *Hemophilus influenzae*, and Fluoroquinolone-resistant *Shigella* spp. are referred to as Priority 3 pathogens [3]. These resistant pathogens are a threat to global health security because of their potential to cause significant economic and public health problems [4, 5].

One of the effective ways to maximize global health security and preparedness for infectious disease threat is to invest in Global Health and address global health security challenges [6]. Since June 2007, various countries have been putting in place efforts to strengthen their International Health Regulations (IHR) core capacities through the Global Health Security expanded activities. As a way of achieving these objectives, WHO conducted a Joint External Evaluation (JEE) of the IHR core capacities in 2017 in Ghana [7]. Among the key findings identified was the need to strengthen laboratory capacities to improve detection of epidemic prone infectious diseases, improve logistics for surveillance, standardize methods for antimicrobial resistance susceptibility testing and improve collaboration between disease surveillance officers and laboratory scientists.

However, availability of human and material resources to address these gaps has been difficult because of funding limitations. Bacterial isolation and identification from clinical specimens such as blood, stool and urine involve the use of sophisticated devices and specialized skills which are expensive [8]. Interventions from partner organizations and non-governmental organizations (NGOs) and/or corporate institutions are needed in the quest to address global health security challenges especially in resource poor settings such as Ghana.

As a way of addressing these challenges, Ghana received funding as one of the high-risk non-Ebola affected countries to strengthen the public health infrastructures and improve detection of epidemic prone infectious pathogens such as *Salmonella, Shigella, Vibrio and* diarrheagenic *E. coli.* The U. S Centers of Disease Control and Prevention engaged the Centre for Health Systems Strengthening (CfHSS) through the Association of Public Health Laboratories (APHL) to design and implement strategies for strengthening Public Health Laboratories (PHLs) in Ghana. This report presents a series of activities leading to improved detection of bacterial pathogens in the laboratory.

## Methods

### Study setting and design

**Fig. 1:**
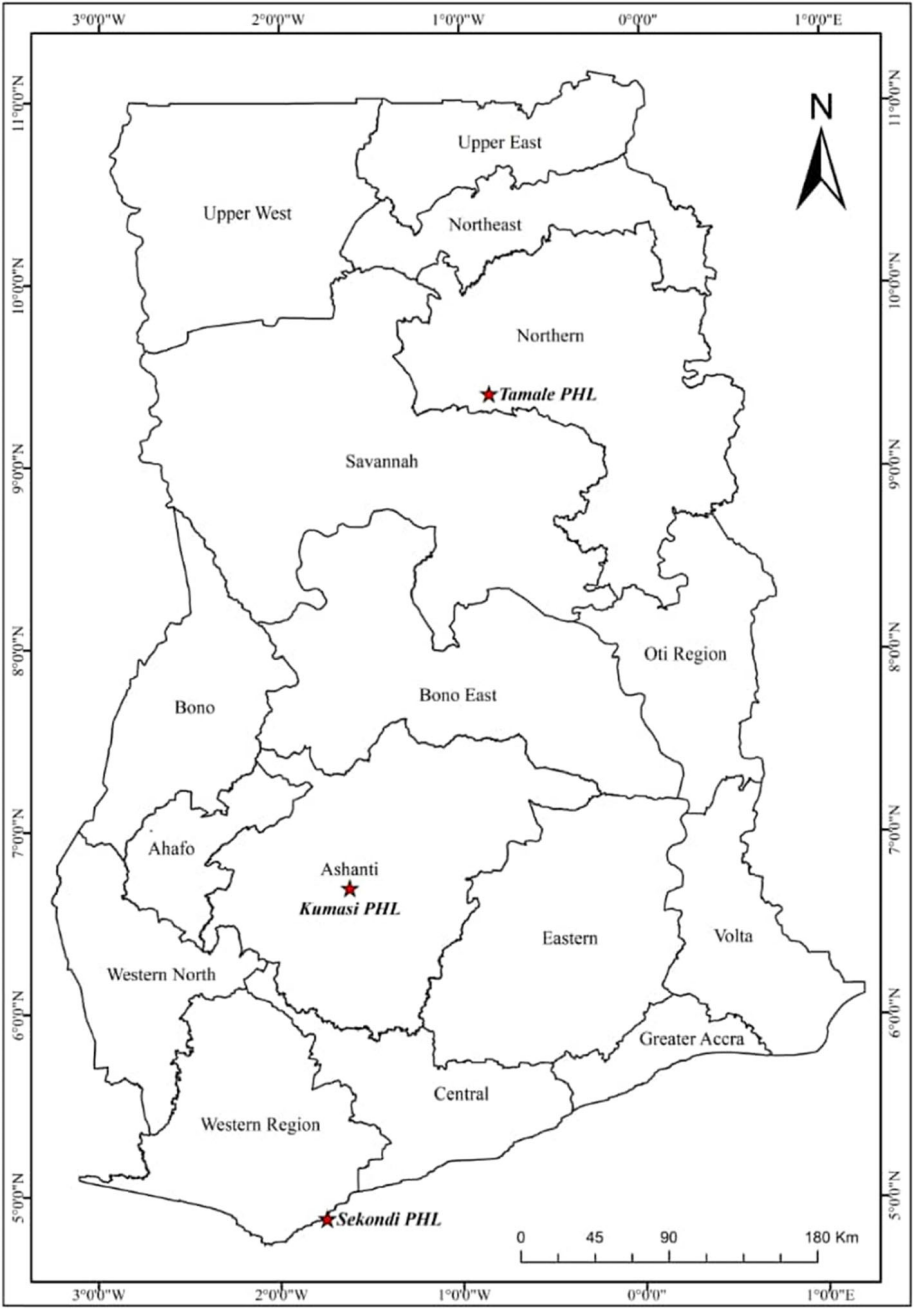
Zonal Public Health Laboratories (black dots) selected for capacity building

This was a multicentric-single country retrospective study conducted between June, 2018 and August, 2019. The PHLs supported were the Tamale Public Health Laboratory (TPHL), Kumasi Public Health Laboratory (KPHL) and Sekondi Public Health Laboratory (SPHL). These three laboratories are strategically located to serve the three zonal sectors of Ghana (Fig. 1). KPHL serves the Ashanti region and other southern parts of Ghana. TPHL serves the five northern regions (Savannah, North-East, Northern, Upper East and Upper West) in Ghana and SPHL serves the Western and Central regions of Ghana. All three laboratories are situated on the premises of tertiary health facilities: KPHL is located on the premises of Ashanti regional hospital in Kumasi; TPHL is located very close to the Tamale Teaching Hospital; and SPHL is found on the same environment as the Effia-Nkwanta regional hospital. The laboratories work closely with these hospitals, and invasive procedures such as lumbar punctures are performed by trained clinicians and physician assistants at the various hospitals and samples transported on ice packs to the PHL for laboratory testing. These laboratories are the first point of call during outbreak situations and they perform range of diagnostic testing, but with limited capacity and infrastructure.

### Study population

Study population comprised individuals of all ages and gender who attended the various hospitals and exhibited clinical presentations of sepsis, gastroenteritis, urinary tract infection and meningitis. Blood and stool cultures were requested by clinicians from individuals with presumptive symptoms of sepsis and gastroenteritis [9]. Urine was collected from individuals who presented with symptoms such as frequent painful urination, hematuria and cloudy/foul-smelling urine for urine culture. Patients with classical case definition for meningitis were referred to trained clinicians for lumbar puncture. The clinical criteria required for lumbar puncture to be performed comprised sudden onset of fever (axillary: > 38 °C), and at least two of the following clinical symptoms: neck pain, neck stiffness, photophobia, reduced level of consciousness, bulging fontanelle, and fits/partial seizures in children between 6 months and 5 years [9, 10].

### Quality assessment prior to start of study

Prior to initiating the programme, we conducted laboratory assessments of the three PHLs using the WHO/CDS/CSR/ISR/2001.2 assessment tool. The assessment evaluated areas including availability of microbiological equipment, scope of laboratory investigations, specimen transportation and handling, adequacy of standard operating procedures (SOPs), internal and external quality assurance and general work flow.

Based on findings from the assessments, in-house mentors and consultants were recruited by CfHSS to assist with capacity building in microbiological investigations and quality management system (QMS). Consultants and mentors assisted in review of SOPs of the laboratories, training laboratory staff on new microbiological techniques such as use of Analytical Profile Index (API), standardizing methods of media preparation and antimicrobial susceptibility testing, establishing sheep farms for blood and chocolate media preparation and training of laboratory staff and disease surveillance officers in the handling, collection and transportation of infectious samples. APHL also established an external quality assurance programme and also supported the procurement of equipment and reagents for smooth operation of the laboratories.

### Ethical approval

Permission was sought from the facilities before collection of this data. All protocols related to data collection and analysis were reviewed and approved by the Ethics Review Committee (ERC) of the Ghana Health Service (GHS) (Approval number: GHS-ERC008/03/20).

### Interventions

As part of the design of this capacity building programme, we focused on improving capacity in the detection of epidemic-prone infectious pathogens such as *Salmonella, Shigella, Vibrio and diarrhegenic E. coli.* Samples such as blood, urine, stool and cerebrospinal fluid (CSF) were given priority. Laboratory staff and disease surveillance officers were adequately trained in collection and transportation of priority specimen from the field to the laboratory under cold chain. As part of the training, SOPs related to specimen transportation, processing and pathogen detection were developed/revised for each of the laboratory sites. QMS were also improved and staff were assigned to specific tasks in line with objectives of the programme.

### Laboratory methods for detection of priority pathogens

Blood samples collected into culture bottles (BD, Franklin Lakes, NJ, USA) were incubated in BACTEC™ 9050 (for TPHL) or BACTEC FX 40 (for SPHL and KPHL) blood culture systems (BD, Franklin Lakes, NJ, USA). Samples were incubated at 35 °C for five days or until a positive signal was detected. Positive blood culture samples were plated on blood and chocolate agar (BD, Franklin Lakes, NJ, USA) and incubated overnight (18–24 h) at 35–37 °C. Urine samples were plated directly onto cysteine lactose electrolyte-deficient (CLED) agar (BD, Franklin Lakes, NJ, USA), and stool samples plated on xylose lysine deoxycholate (XLD), mannitol salt agar (MSA), thiosulphate citrate bile salt sucrose (TCBS), and sorbitol-MacConkey agars (SMAC) (BD, Franklin Lakes, NJ, USA). Both urine and stool samples were incubated overnight (18–24 h) at 35–37 °C.

At all PHL sites, CSF samples were cultured for detection of bacteria pathogens by plating directly on blood and chocolate agars and incubated afterwards at 35–37 °C aerobically and anaerobically, respectively. Gram stain was also performed immediately on all CSF samples for prompt reporting for patient management. At TPHL, multiplex real-time polymerase chain reaction (PCR) was conducted on all CSF samples for further identification and confirmation. Samples were processed after which Mastermix comprising of primers, probes and other reagents were used for simultaneous detection of *Neisseria meningitidis, Haemophilus influenzae and Streptococcus pneumoniae.* One microlitre (1 μl) each of sodC-forward primer, sodC-Reverse primer, sodC-Probe, hpd3-Forward primer, hpd3-Reverse primer, hpd3-Probe, lytA-forward primer, lytA-Reverse primer, lytA-Probe was added to 1.5 μl of PCR grade water, 12.5 μl PCR quantabio and 2 μl of DNA template which resulted in a total reaction volume of 25 μl. PCR amplification was conducted using AriaMx RT-PCR System (Agilent Technologies). Thermal cycling conditions comprised one cycle of initial denaturation at 95 °C for 10 min, followed by 40 cycles of template denaturation at 95 °C for 15 s and annealing at 60 °C for 1 min.

### Bacteria identification

For positive blood culture, a single pure colony was picked from the blood agar for Gram staining. Urine cultures which yielded significant bacteria growth on CLED was selected for Gram staining by picking single well isolated colony. For positive stool culture, colonies which grew on XLD, MSA, TCBS and SMAC agars were sub-cultured onto different blood agars and incubated overnight aerobically. Single pure colonies on the respective blood agars were picked for Gram staining.

Biochemical investigations such as triple sugar iron (TSI), citrate, urease, indole and oxidase tests were performed on all Gram-negative isolates. API 20E and 20NE were also performed on presumptive enterobacteria and non-enterobacterial isolates, respectively. Other tests such as catalase, coagulase and optochin were performed on all Gram-positive bacteria to aid identification of most common Gram-positive pathogens such as *Streptococcus pneumoniae* and *Staphylococcus aureus*.

### Antimicrobial susceptibility testing (AST)

The antibiotics chosen for testing were based on current treatment regimens for Gram-negative and Gram-positive infections in Ghana as well as clinical and laboratory standards institute guidelines (CLSI) [11]. Gram-negative bacteria susceptibility to ampicillin (10 μg), amoxiclav (amoxicillin & clavulanic acid; 20/10 μg), ceftriaxone (30 μg), cefuroxime (30 μg), azithromycin (15 μg), amikacin (30 μg), meropenem (10 μg), trimethoprim/sulfamethoxazole (1.25/23.75 μg), ciprofloxacin (5 μg), gentamicin (10 μg), tetracycline (30 μg), chloramphenicol (30 μg), ceftazidime (30 μg), cefotaxime (30 μg) and nalidixic acid (30 μg) was tested on Mueller-Hinton agar (BD, Franklin Lakes, NJ, USA) using the Kirby-Bauer disc diffusion method. Gram-positive bacteria were also tested against ampicillin (10 μg), amoxiclav (20/10 μg), ceftriaxone (30 μg), azithromycin (15 μg), ciprofloxacin (5 μg), gentamicin (10 μg), tetracycline (30 μg), chloramphenicol (30 μg), cefotaxime (30 μg), amikacin (30 μg) together with erythromycin (15 μg), penicillin (10 units) and clindamycin (2 μg). The breakpoints for the antibiotics used were in line with CLSI 2018 guidelines [11].

### Statistical analysis

Data were collected, entered into Microsoft excel (Microsoft Cooperation, 2013), cleaned and exported to STATA version 12 (Stata Corp, College Station, Texas, USA) for analysis. Descriptive statistics was used to summarize the distribution of various variables into table and graphs. Differences between discrete variables were analyzed using chi-square (or Fisher’s exact where appropriate) and *p* value less than 0.05 was considered statistically significant.

## Results

### Characteristics of study population

A total of 3902 patients were tested in all 3 PHLs of which 2229 (57.1%) were females. The median age of all patients was 18 years (IQR: 3–36 years). There was general increase in total number of samples collected and processed after the programme implementation compared to samples received before implementation across all three PHLs (Table 1). At TPHL, there was statistically significant difference in collection of all samples except blood before and after initiation of programme. Blood, urine and stool were significantly collected at KPHL, whereas in SPHL, only stool specimen was significantly collected before and after programme initiation.

**Table 1:** Distribution of sample types collected at the various PHLs

	TPHL [n (%)]			KPHL [n (%)]			SPHL [n (%)]		
	Before (838)	After (961)	*p-value*	Before (394)	After (705)	*p-value*	Before (1621)	After (2236)	*p-value*
Blood	78 (9.3)	115 (12.0)	0.07	90 (22.8)	99 (14.0)	< 0.001	679 (41.9)	893 (39.9)	0.224
Urine	126 (15.0)	96 (10.0)	0.001	243 (61.7)	509 (72.2)	< 0.001	855 (52.7)	1137 (50.8)	0.245
Stool	80 (9.5)	26 (2.7)	< 0.001	61 (15.5)	39 (5.5)	< 0.001	66 (4.1)	184 (8.2)	< 0.001
CSF	554 (66.1)	724 (75.3)	< 0.001	–	58 (8.2)	–	21 (1.3)	22 (1.0)	0.363

‘Before’ refers to number of samples which arrived at the various PHLs before implementation of the programme; ‘After’ refers to number of samples which arrived at the various PHLs after implementation of the programme

### Sampling trend

The number of blood samples collected in June, 2018 (inception of study) was the lowest (39/1107; 3.5%) recorded throughout the study period. Sample collection improved with time and remained fairly constant till the end of the study (August 2019), with average number of blood samples collected per month to be 74 (6.7%).

Urine was the most collected sample (1742/3903; 44.6%) among all the specimens. The average number of urine samples collected for each month was 116 (6.6%). Generally, there was a downward trend of urine samples collected from November 2018 to March 2019 (Dry Season), with December 2018 recording the least number (77/1743; 4.4%) collected.

**Fig. 2:**
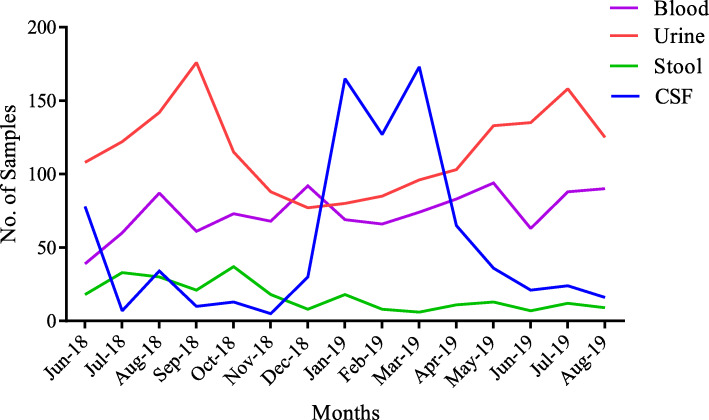
A description of the sampling trend for the duration of this project (June 2018–August 2019)

Total number of stool samples collected over the study period was comparatively low (249/3903; 6.4%), with an average of 17 samples collected every month. There was a sharp rise in CSF samples collected from January 2019 to March 2019 (Dry Season) (Fig. 2).

### Bacterial pathogen distribution

Bacterial pathogens were detected in five hundred and ninety-three (593) out of 3902 clinical specimens. Generally, before programme initiation, there were low proportions of bacterial isolates recovered from the clinical specimens across all three PHLs (except for bacteria from CSF identified by PCR at TPHL) as shown in Table 2. Of the 593 isolates, bacterial pathogens were identified in 70 (11.8%) blood cultures, 356 (60%) urine cultures, 19 (3.2%) stool cultures and 148 (25%) CSF samples after programme implementation (Table 2). There were more bacteria isolated from blood and urine after programme implementation compared to before at SPHL and this difference was statistically significant. Bacteria isolated from urine after programme implementation was disproportionately higher than before but the difference was not significant. After implementation, only 6 (0.1%) samples were contaminated with Coagulase-negative Staphylococci (CNS) and these emanated from blood (2) and urine (4).

**Table 2:** Distribution of bacterial pathogens among the study sites

	TPHL [n (%)]			KPHL [n (%)]			SPHL [n (%)]		
	Before (192)	After (203)	*p-value*	Before (36)	After (175)	*p-value*	Before (96)	After (215)	*p-value*
Blood	2 (1.0)	17 (8.4)	< 0.001	0	10 (5.7)	0.14	1 (1.0)	43 (20.0)	< 0.001
Urine	14 (7.3)	41 (20.2)	< 0.001	36	156 (89.1)	0.04	91 (94.8)	159 (74.0)	< 0.001
Stool	7 (3.6)	0	0.01	0	7 (4.0)	0.22	2 (2.1)	12 (5.6)	0.169
CSF	169 (88.0)	145 (71.4)	< 0.001	–	2 (1.1)	–	2 (2.1)	1 (0.5)	0.177

‘Before’ refers to number of bacterial pathogens identified at the various PHLs before implementation of the programme; ‘After’ refers to number of bacterial pathogens which were indentified at the various PHLs after implementation of the programme

The most predominant bacterial pathogen isolated from blood was *Staphylococcus aureus* (22/70; 31%) (Table 3). *Escherichia coli* was commonly found in the urine samples (153/356; 43%) and *Vibrio parahaemolyticus* was high in stool (5/19; 26.3%). There were 3 main pathogens identified in the CSF samples - *Neisseria meningitidis*, *Streptococcus pneumoniae* and *Hemophilus influenzae*, with *S. pneumoniae* being the most isolated pathogen (80/148; 54.1%).

**Table 3:** Laboratory investigation on results from bacterial isolates

Laboratory results	Study sites			
	TPHL	KPHL	SPHL	Total
Blood culture (BC) results				
Total BC performed	115	99	893	1107
Total BC pathogens	17	10	43	70
*Escherichia coli*	3	3	3	9
*Klebsiella pneumoniae*	4	2	2	8
*Klebsiella aerogenes*	0	1	0	1
*Klebsiella spp.*	0	1	4	5
*Pseudomonas aeruginosa*	1	2	2	5
*Salmonella spp.*	0	0	5	5
*Salmonella* Typhi	0	0	8	8
*Staphylococcus aureus*	9	0	13	22
Others	0	1	6	7
Urine culture (UC) results				
Total UC performed	96	509	1137	1742
Total UC pathogens	41	156	159	356
*Escherichia coli*	16	63	74	153
*Pseudomonas* spp.	0	11	0	11
*Pseudomonas aeruginosa*	0	7	3	10
*Proteus vulgaris*	1	4	1	6
*Proteus mirabilis*	0	5	0	5
*Proteus* spp.	0	4	0	4
*Klebsiella pneumoniae*	5	8	0	13
*Klebsiella oxytoca*	0	1	0	1
*Klebsiella* spp.	0	27	32	59
*Citrobacter* spp.	1	3	21	25
*Staphylococcus aureus*	8	4	4	16
*S. saprophyticus*	1	10	11	22
Others	9	11	13	33
Stool culture (SC) results				
Total SC performed	26	39	184	249
Total SC pathogens	0	7	12	19
EHEC O157:H7	0	3	0	3
*Staphylococcus aureus*	0	2	0	2
*Shigella* spp.	0	0	3	3
*Vibrio parahaemolyticus*	0	0	5	5
Others	0	2	4	6
Cerebrospinal fluid (CSF) PCR results				
Total PCR performed	724	58	22	804
Total pathogens	145	2	1	148
*Neisseria meningitidis*	61	1	1	63
*Streptococcus pneumoniae*	80	1	0	81
*Hemophilus influenzae*	10	0	0	10

*S. aureus* was commonly isolated from the blood of patients in SPHL (*n* = 13), followed by TPHL (*n* = 9), with no isolation from KPHL. *E. coli* causing urinary tract infection (UTI) were mostly isolated from individuals in SPHL (*n* = 74), followed by KPHL (*n* = 63) and then TPHL (*n* = 16). Isolation of bacteria causing meningitis was extremely high in CSF specimens of patients who attended TPHL (151/154; 98.1%). We observed that new pathogens such as *Pastuerella pneumotropica, Klebsiella oxytoca, Vibrio parahaemolyticus, Enterobacter aerogenes, Halfnia alvei, Serratia odonfera1* and *Citrobacter freundii* were identified with the aid of API. Hitherto, these pathogens had never been isolated in any of the three PHLs.

### Seasonal variations in prevalence of bacterial pathogens

**Fig. 3:**
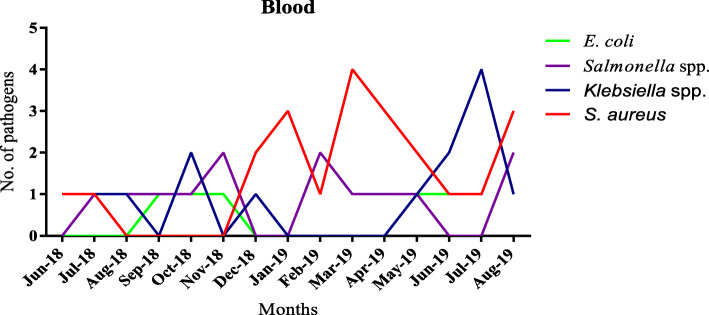
Seasonality of blood culture isolates

**Fig. 4:**
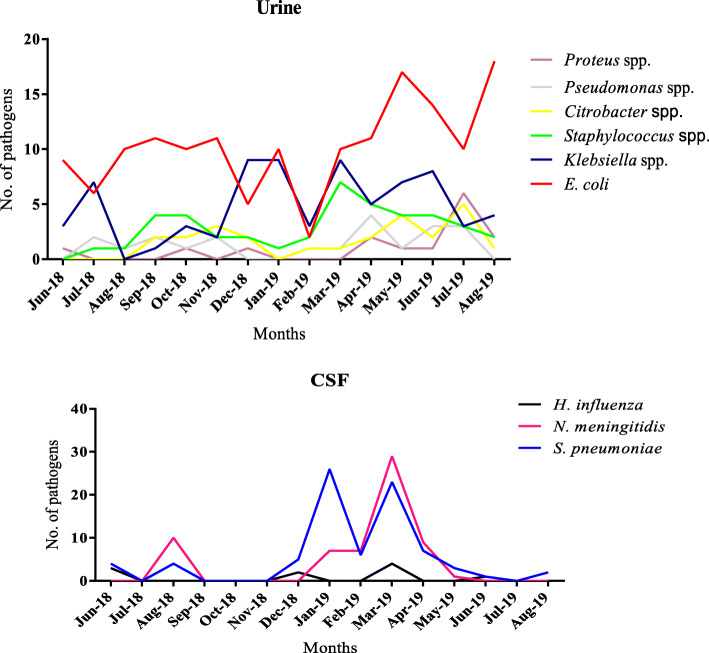
Seasonality of isolates in Urine and CSF

Ghana has two seasons: dry and wet seasons. The wet season is from April to mid-October and the dry season is from December to March. Detection of pathogens from the blood during the first four months of the study was relatively lower compared to the months afterwards (Fig. 3). There was high isolation of *S. aureus* and *Salmonella spp.* from blood samples in the dry season. The wet season mostly had *Klebsiella* spp. been isolated*.* There was a steady rise in *Streptococcus pneumoniae* and *Neisseria meningitidis* detection from CSF in the dry season. *Streptococcus pneumoniae* isolates mostly occurred in January and co-detections of *Streptococcus pneumoniae* and *Neisseria meningitidis* occurred in March (Fig. 4). Urinary pathogens did not show any particular seasonality. However, high isolations of *E. coli* occurred mostly in the wet season as compared to the dry season (Fig. 4).

### Effects of socio-demographics on bacterial infection

Females were more likely to contract urinary tract infection than males (Table 4; *p* < 0.01). Nonetheless, gender did not significantly contribute to the likelihood of having bacterial infection from blood, stool and/or CSF. Again, children less than 18 years were more prone to bacterial infection in the blood and CSF than the adults (Table 4). On the other hand, urinary tract infection (UTI) in adults were significantly higher than in children.

**Table 4:** Bacterial infections according to age and gender

	Sample [n (%)]			
Variable	Blood	Urine	Stool	CSF
**Sex**				
M	35 (50)	95 (26.5)	7 (36.8)	79 (54.1)
F	35 (50)	263 (73.5)	12 (63.2)	67 (45.9)
*p-value*	0.51	< 0.01	0.65	0.21
**Age (years)**				
0–5	16 (22.9)	64 (17.9)	2 (10.5)	25 (16.9)
6–18	9 (12.9)	47 (13.1)	0	46 (31.1)
> 18	45 (64.2)	247 (69)	17 (89.5)	77 (52)
*p-value*	0.52	0.07	0.15	0.62

### Antimicrobial susceptibility results

#### Blood

Overall, gentamicin was the least effective antibiotic with 35–55% of *S. aureus*, *K. pneumoniae* and *E. coli* bacteria resistant to this antibiotic (Table 5). *Salmonella* spp. showed high proportion of resistance to ampicillin, cefuroxime and ciprofloxacin (38.5%) but not to azithromycin, tetracycline, and cefotaxime (7.7%).

**Table 5:** Antimicrobial susceptibility patterns of most common bacterial pathogens

Proportion of resistant bacterial species [n (%)]												
	Blood					Urine						
	*S. aureus* (*n* = 22)	*Klebsiella* spp. (*n* = 14)	*Salmonella* spp. (n = 13)	*E. coli* (n = 9)	*p-value*	*E. coli* (*n* = 153)	*Klebsiella* spp. (*n* = 73)	*Staphylococcus* spp. (*n* = 41)	*Citrobacter* spp. (*n* = 25)	*Pseudomonas* spp. (*n* = 21)	*Proteus* spp. (n = 15)	*p-value*
AMP	4 (18.2)	6 (42.9)	5 (38.5)	2 (22.2)	0.348	52 (34.0)	18 (24.7)	4 (9.8)	8 (32.0)	–	1 (6.7)	0.006
AMC	1 (4.5)	1 (7.1)	0	0	0.696	16 (10.5)	7 (9.6)	3 (7.3)	7 (28.0)	3 (14.3)	1 (6.7)	0.084
CRO	2 (9.1)	4 (28.6)	2 (15.4)	3 (33.3)	0.314	49 (32.0)	23 (31.5)	8 (19.5)	9 (36.0)	7 (33.3)	5 (33.3)	0.671
CXM	–	2 (14.3)	5 (38.5)	0	0.074	41 (26.8)	17 (23.3)	2 (4.9)	5 (20.0)	6 (28.6)	4 (26.7)	0.042
AZM	2 (9.1)	0	1 (7.7)	–	0.607	15 (9.8)	3 (4.1)	5 (12.2)	1 (4.0)	–	1 (6.7)	0.460
MEM	–	0	–	–	–	0	0	–	0	2 (9.5)	0	0.008
SXT	0	1 (7.1)	0	0	0.621	8 (5.2)	6 (8.2)	3 (7.3)	2 (8.0)	2 (9.5)	0	0.779
CIP	6 (27.3)	2 (14.3)	5 (38.5)	4 (44.4)	0.360	60 (39.2)	22 (30.1)	16 (39.0)	13 (52.0)	4 (19.0)	6 (40.0)	0.200
GEN	12 (54.5)	5 (35.7)	3 (23.1)	4 (44.4)	0.314	42 (27.5)	23 (31.5)	12 (29.3)	9 (36.0)	4 (19.0)	4 (26.7)	0.849
TET	6 (27.3)	1 (7.1)	1 (7.7)	–	0.219	23 (15.0)	12 (16.4)	3 (7.3)	1 (4.0)	1 (4.8)	–	0.293
C	2 (9.1)	0	0	0	0.641	4 (2.6)	2 (2.7)	3 (7.3)	0	1 (4.8)	–	0.437
CAZ	–	1 (7.1)	–	–	–	4 (2.6)	4 (5.5)	0	–	1 (4.8)	3 (20.0)	0.026
CTX	2 (9.1)	3 (21.4)	1 (7.7)	3 (33.3)	0.302	33 (21.6)	29 (39.7)	9 (22.0)	4 (16.0)	11 (52.4)	7 (46.7)	0.002
NAL	–	1 (7.1)	–	2 (22.2)	0.538	42 (27.5)	16 (21.9)	–	4 (16.0)	8 (38.1)	7 (46.7)	0.159
ERY	0	–	–	–	–	–	–	8 (19.5)	–	–	–	–
PEN	–	–	–	–	–	–	–	–	–	–	–	–
CLI	2 (9.1)	–	–	–	–	–	–	3 (7.3)	–	–	–	–
AMK	0	1 (7.1)	3 (23.1)	1(11.1)	0.071	23 (15.0)	13 (17.8)	6 (14.6)	5 (20.0)	2 (9.5)	0	0.550

AMP - ampicillin, AMC - amoxicillin & clavulanic acid, CRO - ceftriaxone, CXM - cefuroxime, AZM - azithromycin, AMK - amikacin, MEM - meropenem, SXT - trimethoprim/sulfamethoxazole, CIP - ciprofloxacin, GEN - gentamicin, TET - tetracycline, C - chloramphenicol, CAZ - ceftazidime, CTX - cefotaxime and NAL - nalidixic acid, ERY - erythromycin, penicillin and CLI - clindamycin

#### Urine

*E. coli* recorded high resistance to ciprofloxacin (39.2%) and ampicillin (34.0%) (Table 5). *Klebsiella* spp. was highly resistant to the 3rd generation cephalosporin cefotaxime (39.7%), followed by ceftriaxone (31.5%) and then gentamicin (31.5%). Strains of *Staphylococcus* spp. and *Citrobacter* spp. were found to show high resistance to ciprofloxacin (39 and 52%, respectively). Half of the *Pseudomonas* spp. isolated from urine were resistant to cefotaxime. Likewise, almost half of *Proteus* spp. found in urine were resistant to cefotaxime and nalidixic acid (46.7%) (Table 5).

## Discussion

The role microbiological laboratories play in the detection and surveillance of pathogenic bacteria is important in addressing the global health security threats posed by infectious agents. Resourcing of the PHLs with equipment, reagents and human resource capacity building have enabled the increased and accurate detection of bacterial pathogens from clinical specimens of blood, stool, urine and cerebro-spinal fluid at three different PHLs in Ghana. It is instructive to note that prior to this programme, TPHL and KPHL for instance had stopped blood cultures due to unavailability of logistics and non-functional BACTEC equipment. SPHL as well was not well-versed in the use of CLSI standards for isolation of bacteria. Adequate measures including provision of distilled water plants, establishments of sheep farms, training in microbiological media preparation and identification of microbial pathogens were put in place. Training and adequate resourcing are therefore essential in strengthening the capacity and functional role of PHLs in developing countries.

In sub-Saharan Africa (sSA), about 12 million people die each year [12], with the causes of deaths largely due to undiagnosed infectious diseases such as HIV, malaria and tuberculosis [13]. A study in Kenya found that bacterial bloodstream infection diagnosed only by blood culture accounted for 26% of deaths among children [14], which gives credence to the fact that laboratory diagnosis of bacterial infections needs to be strengthened in sub-Sahara Africa. In line with previous studies in Africa [15, 16], Gram-negative bacteria predominated in our study. Infections from Gram-negative bacteria pose significant public health problems and this is mainly due to high resistance to antimicrobial agents [17]. Prior to implementation of the project, all three PHLs had serious challenges with regards to microbiological detection of bacterial pathogens from clinical specimens. At TPHL, identification was solely done by observing morphological characteristics of the colonies which grew on various culture media. There was absence of biochemical testing and automated blood culture system due to logistical and technical constraints. Both KPHL and SPHL performed very limited biochemical tests, and only SPHL performed automated blood culture analysis. Across the three PHLs, identification of suspected bacterial pathogens was performed up to the Genus level. All these hindered the identification process and proper administration of antimicrobials, which might directly or indirectly contribute to increasing rate of antimicrobial resistance globally. Procurement and maintenance of automated blood culture machines for the PHLs and training of staffs on its use resulted in an increased rate of blood samples received for blood culture. Reagents necessary for biochemical tests were also procured for all the PHLs and staffs were trained on the use and interpretation of tests such as triple sugar iron (TSI), citrate, indole, catalase, urease, coagulase and catalase tests. Analytical profile index and serotyping were also introduced to all PHLs which served as confirmatory tests for identification of some microbial pathogens. All these led to a significant upsurge in bacterial detection (Table 3) which hitherto could have easily been overlooked and missed.

*Staphylococcus aureus* was the most prevalent bacteria found in the blood. Naber reported that *S. aureus* is a major cause of bacteremia, and it is associated with higher morbidity and mortality, compared with bacteremia caused by other pathogens [18]. Again, life-threatening complications from *S. aureus* bloodstream infections such as infective endocarditis and metastatic infections could occur [19, 20], and these complications place high resource burden on health-care systems [21]. Without quality laboratory testing, detection of this medically relevant organism could not be done, and this would have a devastating clinical outcome on the patients. However, in this crisis time, laboratory and healthcare infrastructures are woefully inadequate to meet the pressing needs and/or perhaps have been ignored in several areas of sSA [22]. More often than not, a lot of financial resources from funding organizations are channeled to prevention of diseases and patients’ care, whereas building of laboratory capacity receives relatively little financial support [23].

*E. coli* and *V. parahaemolyticus* were commonly isolated pathogens from urine and stool cultures, respectively. Other common pathogens detected in urine included *Klebsiella* spp., *Citrobacter* spp., *Staphylococcus saprophyticus* and *Staphylococcus aureus.* UTI is among the leading causes of morbidity and mortality and inappropriate diagnosis could lead to treatment failures and additional complications [24]. This study showed that females were more likely to contract UTI than males, consistent with findings from rural Nigeria [25] and other parts of the world [26, 27]. In women, UTIs are one of the most frequent clinical bacterial infections, constituting about 25% of all infections [28]. This is mainly due to women having extremely short urethra, very close to the anal region, where several enterobacteria are shed frequently in stool. Other factors thought to predispose women to recurrent UTIs include voiding patterns pre- and post-coitus, wiping techniques, wearing tight undergarments, and vaginal douching; however, there is no proven association [29]. Also, medical conditions such as pregnancy, diabetes mellitus and immunosuppression increase risk of women having UTI [30]. Cases of *V. parahaemolyticus* infection are few globally; however, it is a common cause of bacterial gastroenteritis in Asia, especially in Japan [31]. Transmission of *V. parahaemolyticus* is mainly through the consumption of infected seafood causing acute gastroenteritis [32]. The organism can also make its way into an open wound during exposure to salt water [31]. All *V. parahaemolyticus* cases recorded in this study were from SPHL; located in the only coastal and southernmost region in this study where consumption of raw/undercooked seafoods is high. SPHL received the most specimens, because of its strategic location within the enclave of the South-Western part of Ghana. As a result, clinicians could easily refer patients to the laboratory for culture analysis. Inhabitants living along the coast principally engage in numerous outdoor activities such as fish farming and swimming in the deep ocean which predispose them to some waterborne infections, especially urinary tract infections (UTIs), and this could also account for the high proportions of urine samples collected in SPHL compared to the other PHLs.

Most detections of *S. pneumoniae, H. influenzae* and *N. meningitidis* in this study were achieved by multiplex real-time PCR. This platform was however only available at the TPHL, which also doubles as a reference testing centre for meningitis in Ghana. It is possible the other laboratories had low detections because of the use of only culture methods which is less sensitive as compared to multiplex PCR. PCR is a fast, sensitive and reliable technique for simultaneous detection of different molecular targets in one reaction. Training and logistical support provided by the CDC to the TPHL enabled them to utilize this molecular approach to detect common etiological agents of meningitis from CSF in Ghana. It would be important for the other PHLs to be adequately resourced with molecular testing capacities to help them to appropriately detect and respond to infectious agents and outbreaks.

The high number of meningitis pathogens identified at the TPHL could also be due to the geographical location and catchment populations targeted which lies within the sSA meningitis belt. Cases of meningitis are frequently reported from the Northern part of Ghana, where TPHL is situated, especially during the hot dry seasons (December – March, and August), leading to several meningitis outbreaks. Unlike CSF samples, other specimens such as urine, stool and blood did not show such seasonal trend. It is an established fact that cerebrospinal meningitis is an infectious disease which is commonly impacted by climate, specifically hot climate [10]. The five Northern regions (Upper East, Upper West, Savannah, North-East and Northern regions) are the most hottest regions in Ghana and the weather worsens during the period between December and June, and this results in a number of CSM outbreaks in Northern Ghana, as previously reported [10, 33]. Several studies have indicated that climatic conditions characterized by dry winds, dust storms, low humidity and cold nights considerably diminish the local immunity of the pharynx thereby increasing the risk of meningitis [34–36]. These climatic conditions are typically found in the Northern Ghana during the dry season, and this likely explains why CSF samples were largely collected during the peak dry seasons compared to the other sample types.

Almost all CSF pathogens (145/148; 98%) recorded in this study emanated from TPHL. *Streptococcus pneumoniae* was the most isolated pathogen from CSF, consistent with previous data from Brong Ahafo region [37], about 210 km away from Tamale. This Global Health Security pathogen is the leading cause of bacterial meningitis, with an average mortality rate of 25%, despite effective antibiotic therapy and improved intensive care facilities [38]. Bacterial meningitis outbreaks are common in countries located in the Africa’s meningitis belt [39]. Rapid detection of the etiology of these outbreaks can lead to targeted public health interventions. Building and sustaining laboratory capacity in countries where meningitis outbreaks are common will be critical to ensure rapid and effective response to these outbreaks.

Global emergence and spread of antibiotic resistant strains of bacteria is still a major health problem. CfHSS provided training in the performance and interpretation of antimicrobial susceptibility tests to all the PHLs. Guidelines and protocols by Clinical and Laboratory Standards Institute (CLSI) were made available to the PHLs and all staff were adequately trained in the use of these documents. Antimicrobial susceptibility tests from blood cultures in this study revealed high resistance (35–55%) of *S. aureus*, *K. pneumoniae* and *E. coli* to gentamicin. This drug is commonly used to treat wide range of Gram-negative and some Gram-positive infections due to its antimicrobial efficacy, widespread availability and low cost [40]. However, the present report shows increase in resistance to this vital drug, consistent with data reported by Ababneh and colleagues [41]. *Escherichia coli* in urine showed high resistance to drugs such as ciprofloxacin (39.2%) and ampicillin (34%). These are historically useful antibiotics for the treatment of UTIs [42]. Also, high rate of *Klebsiella* spp. resistance (39.7%) to the 3rd generation cephalosporin cefotaxime is of great concern. Resistance to 3rd generation cephalosporins such as cefotaxime, ceftazidime and ceftriaxone serves as surrogate marker for detection of extended-spectrum beta-lactamases (ESBL) [43]. There have been reported cases of increasing trend of *Klebsiella* resistance to these 3rd generation cephalosporins [44, 45]. Third generation cephalosporin resistance leaves clinicians with limited options for treating patients with gram-negative infections, and as a result, relatively expensive drugs within the carbapenem class are usually considered the treatment of choice [45].

A limitation of this study was the inability to examine for the presence of ESBL phenotypes in the bacterial isolates which were resistant to the 3rd generation cephalosporins. A suggested approach would be to conduct double-disc diffusion synergy test for phenotypic confirmation of organisms possibly encoding ESBL. Another limitation was the exclusion of laboratory detection of viral and parasitic infections. This was due to restricted CDC-Ghana PHL budget for the project. Further support is therefore needed for capacity building in the detection of viruses and parasites in the three zonal PHLs.

## Conclusion

Isolation and proper identification of aetiological agents in bacterial infection are of great importance. Partner and NGOs are encouraged to contribute their quota to help strengthen PHLs in sub-Saharan Africa. Outcome of this report clearly indicates that with routine and effective laboratory trainings, equipment and reagents support, detection of bacteria pathogens could be greatly enhanced, and the right antimicrobial therapy administered.

## Abbreviations

GHSA: Global Health Security Agenda
IHR: International Health Regulations
AST: Antimicrobial Susceptibility Testing
PHL: Public Health Laboratories
CDC: Centers for Disease Control and Prevention
APHL: Association of Public Health Laboratories
CfHSS: Centre for Health System Strengthening
NGO: Non-Governmental Organization
WHO: World Health Organization
SOP: Standard Operating Procedure
QMS: Quality Management System
API: Analytical Profile Index
ERC: Ethics Review Committee
GHS: Ghana Health Service
CLED: Cysteine Lactose Electrolyte-Deficient
XLD: Xylose Lysine Deoxycholate
MSA: Manitol Salt Agar
TCBS: Thiosulphate Citrate Bile Salt Sucrose
SMAC: Sorbitol-MacConkey Agar
PCR: Polymerase Chain Reaction
